# The role of virtual reality in improving motor performance as revealed by EEG: a randomized clinical trial

**DOI:** 10.1186/s12984-017-0268-4

**Published:** 2017-06-07

**Authors:** Rocco Salvatore Calabrò, Antonino Naro, Margherita Russo, Antonino Leo, Rosaria De Luca, Tina Balletta, Antonio Buda, Gianluca La Rosa, Alessia Bramanti, Placido Bramanti

**Affiliations:** IRCCS Centro Neurolesi “Bonino-Pulejo”, Messina, Italy

**Keywords:** Lokomat, Ersp, Loreta, Mirror neuron system, Virtual reality

## Abstract

**Background:**

Many studies have demonstrated the usefulness of repetitive task practice by using robotic-assisted gait training (RAGT) devices, including Lokomat, for the treatment of lower limb paresis. Virtual reality (VR) has proved to be a valuable tool to improve neurorehabilitation training. The aim of our pilot randomized clinical trial was to understand the neurophysiological basis of motor function recovery induced by the association between RAGT (by using Lokomat device) and VR (an animated avatar in a 2D VR) by studying electroencephalographic (EEG) oscillations.

**Methods:**

Twenty-four patients suffering from a first unilateral ischemic stroke in the chronic phase were randomized into two groups. One group performed 40 sessions of Lokomat with VR (RAGT + VR), whereas the other group underwent Lokomat without VR (RAGT-VR). The outcomes (clinical, kinematic, and EEG) were measured before and after the robotic intervention.

**Results:**

As compared to the RAGT-VR group, all the patients of the RAGT + VR group improved in the Rivermead Mobility Index and Tinetti Performance Oriented Mobility Assessment. Moreover, they showed stronger event-related spectral perturbations in the high-γ and β bands and larger fronto-central cortical activations in the affected hemisphere.

**Conclusions:**

The robotic-based rehabilitation combined with VR in patients with chronic hemiparesis induced an improvement in gait and balance. EEG data suggest that the use of VR may entrain several brain areas (probably encompassing the mirror neuron system) involved in motor planning and learning, thus leading to an enhanced motor performance.

**Trial registration:**

Retrospectively registered in Clinical Trials on 21-11-2016, n.NCT02971371.

## Background

Virtual reality (VR) is the simulation of a real environment generated by a computer software and experienced by the user through a human–machine interface [1]. This interface enables the patient to perceive the environment as real and 3D (i.e., the sense of presence), thus increasing patient’s engagement (i.e., embodiment) [2]. Hence, VR can be used to provide the patient with repetitive, task-specific training (as opposed to simply using a limb by chance) that are effective for motor learning functions [3–6]. In fact, VR provides the patient with multisensory feedbacks that can potentiate the use-dependent plasticity processes within the sensory-motor cortex, thus promoting/enhancing functional motor recovery [7–14]. Furthermore, VR can increase patients’ motivation during rehabilitation by decreasing the perception of exertion [8], thus allowing patients to exercise more effortlessly and regularly [9].

It is possible to magnify the sense of presence by manipulating the characteristics of the VR, including screen size, duration of exposure, the realism of the presentation, and the use of animated avatar, i.e., a third-person view of the user that appears as a player in the VR [15]. About that, the use of an avatar may strengthen the use-dependent plastic changes within higher sensory-motor areas belonging to the mirror neuron system (MNS) [16–18]. In fact, the observation of an action, even simulated (on a screen, as in the case of VR), allows the recruitment of stored motor programs that would promote, in turn, movement execution recovery [19, 20]. These processes are expressed by wide changes in α and β oscillation magnitude at the electroencephalography (EEG) (including an α activity decrease and a β activity increase) across the brain areas putatively belonging to the MNS (including the inferior frontal gyrus, the lower part of the precentral gyrus, the rostral part of the inferior parietal lobule, and the temporal, occipital and parietal visual areas) [8, 9, 21, 22].

In the last years, motor function recovery has benefited from the use of robotic devices. In particular, robot-assisted gait training (RAGT) provides the patient with highly repeated movement execution, whose feedback, in turn, permits to boost the abovementioned use-dependent plasticity processes [23]. RAGT has been combined with VR to further improve gait in patients suffering from different neurologic diseases [24]. Nonetheless, the knowledge of the neurophysiologic substrate underpinning neurorobotic and VR interaction is still poor [25, 26]. Indeed, a better understanding of this interaction would allow physician to design more personalized rehabilitative approaches concerning the individual brain plasticity potential to be harnessed to gain functional recovery [27].

The relative suppression of the μ rhythm is considered as the main index of MNS activity [28]. Nonetheless, conjugating VR and neurorobotic could make brain dynamics more complex, because of many factors related to motor control and psychological aspects come into play, including intrinsic motivation, selective attention, goal setting, working memory, decision making, positive self-concept, and self-control. Altogether, these aspects may modify and extend the range of brain rhythms deriving from different cortical areas related to MNS activation by locomotion, including theta and gamma oscillations [29–31]. Specifically, theta activity has been related to the retrieval of stored motor memory traces, whereas the gamma may be linked to the conscious access to visual target representations [30, 31]. Such broadband involvement may be due to the recruitment of multiple brain pathways expressing both bottom-up (automatic recruitment of movement simulation) and top-down (task-driven) neural processes within the MNS implicated in locomotion recognition [32]. A recent work has shown that observed, executed, and imagined action representations are decoded from putative mirror neuron areas, including Broca’s area and ventral premotor cortex, which have a complex interplay with the traditional MNS areas generating the μ rhythm [33].

Therefore, we hypothesized that the combined use of VR and RAGT may induce a stronger and wider modification of the brain oscillations deriving from the putative MNS areas, thus augmenting locomotor function gain [34, 35]. The aim of our pilot randomized clinical trial was to understand the neurophysiological basis underpinning gait recovery induced by the observation of an animated avatar in a 2D VR while performing RAGT by studying the temporal patterns of broadband cortical activations.

## Methods

### Participants

The present randomized clinical trial was conducted according to the CONSORT guidelines [36]. The trial was designed as a pilot, prospective, assessor blinded, parallel group study, and was performed at the IRCCS Centro Neurolesi “Bonino-Pulejo” (Messina, Italy). Eligible patients were enrolled between October 2015 and February 2016, according to the following criteria: (i) age ≥ 55 years; (ii) a first-ever ischemic supra-tentorial stroke (confirmed by magnetic resonance imaging -MRI) at least 6 months before their enrollment; (iii) an unilateral hemiparesis, with a Muscle Research Council -MRC- score ≤ 3 (a score of 3 indicates that muscle strength is reduced so that the joint can be moved only against gravity with the examiner’s resistance completely removed; 2 = muscle can move only if the resistance of gravity is removed; 1 = only a trace of movement; 0 = no movement observed) [37]; (iv) ability to follow verbal instructions, with a Mini-Mental State Examination >24; (v) a mild to moderate spasticity of muscles of hip, knee, and ankle (according to a Modified Ashworth Scale, MAS, ≤2) [38]; (vi) ability to perform manual gait training with or without external devices (Functional Ambulatory Categories 0-4). (vii) no severe bone or joint disease; and (viii) no history of concomitant neurodegenerative diseases or brain surgery.

We preferred to select patients with a first-ever ischemic supra-tentorial stroke, as this represents a better model of stroke lesion to perform EEG analysis. In fact, multiple vascular lesions could represent a significant limit for data interpretation, as they can generate variable signals that can interfere with signal recording. For instance, it has been reported that hemispheric powers differ clearly in single acute ischemic episodes, but correlate less well with the subtle, multifocal, or more gradual changes [39]. Moreover, single lesion model is more suitable to study interhemispheric balance. Last, patients with multiple vascular lesions may have different functional recovery. For the same reason, we limited the age of inclusion to >55, because beneath this age it is necessary to take into account other several, additional risk factors (including hemostatic, inflammatory and autoimmune factors, and cardioembolic sources, such as patent foramen ovale) that altogether increase the risk of multiple vascular lesions and may account for heterogeneity of sample [40].

Clinic-demographic characteristics are reported in Table 1. All participants gave informed consent before study participation. Additionally, written informed consent for publication of clinical images was obtained from the participants. Approval was obtained from our local Ethics Committee before beginning the study (study number registration 43/2013). The study was retrospectively registered in Clinical Trials on 21-11-2016, n.NCT02971371.

**Table 1:** Shows the individual clinical-demographic characteristics. There were no significant between-group differences in any parameter

group	age	gender	lesion location	stroke onset	comorbidities
RAGT + VR	68	M	r FP	12	3
	63	M	l PO	7	2,3
	57	M	r TP	5	1,4
	62	F	l PO	7	1
	60	M	r FP	6	1,2
	59	M	r P	10	2,3
	66	M	l F	6	3
	56	F	r FP	10	1,2
	58	M	l PO	8	1,2,4
	55	F	r FP	8	1,4
	65	F	l PO	8	1,3
	55	F	r TP	7	1,4
mean ± SD	60 ± 4	7 M;5F		8 ± 2	
RAGT-VR	58	M	r P	8	1,3,5
	72	F	l F	11	2,3
	59	M	l F	5	1,2
	54	M	r P	5	2
	55	F	r P	10	1
	73	M	r TP	8	3,5
	63	F	l TP	8	2,2
	64	M	r P	11	2,3
	64	F	l F	6	3
	65	M	r P	8	1,3,5
	65	F	l F	8	2,3
	66	M	l F	8	3
mean ± SD	63 ± 6	7 M;5F		8 ± 2	

*Legend*: *F* frontal; *P* parietal; *O* occipital; *T* temporal; *l* left; *r* right; *1* high blood pressure; *2* diabetes mellitus; *3* hypercholesterolemia; *4* smoking; *5* alcoholism

### Sample size

The sample size estimate was based on extrapolations from previous studies examining the effects of VR on gait in patients with stroke [41–44]. Accordingly, we used the effect size (0.9) of the primary composite endpoint for calculations. Power was set at 80%, alpha at 5%; we accounted for a dropout rate of 10%. Using a relatively conservative estimation, a total of 50 subjects (25 in each group) would be required to detect a difference of at least 20% in the primary outcome between the two treatment groups assuming non-inferiority with moderate correlations among covariates (R-squared = 0.5).

### Study design

Thirty-five patients were assessed for eligibility according to the inclusion/exclusion criteria. Then, 24 patients were equally randomized into the RAGT + VR or RAGT-VR group with a 1:1 allocation ratio. For randomization, sealed envelopes were prepared in advance and marked on the inside with a + VR or -VR. The intervention period of both groups was identical, five sessions per week for eight consecutive weeks, 45-min for each session. We registered the EEG in the morning of the second day of the first week (T_PRE_) and the last day of the last week of treatment (T_POST_). Clinic and kinematic assessment was performed immediately before and after the Lokomat training. In addition, a physiotherapist supervised patients’ cooperation and participation to Lokomat training. The experimenters who analyzed the data and the therapists who performed the clinical tests were blind on patient allocation. Moreover, patients were not informed on the content of the VR adopted in the two groups.

### RAGT intervention

**Fig. 1:**
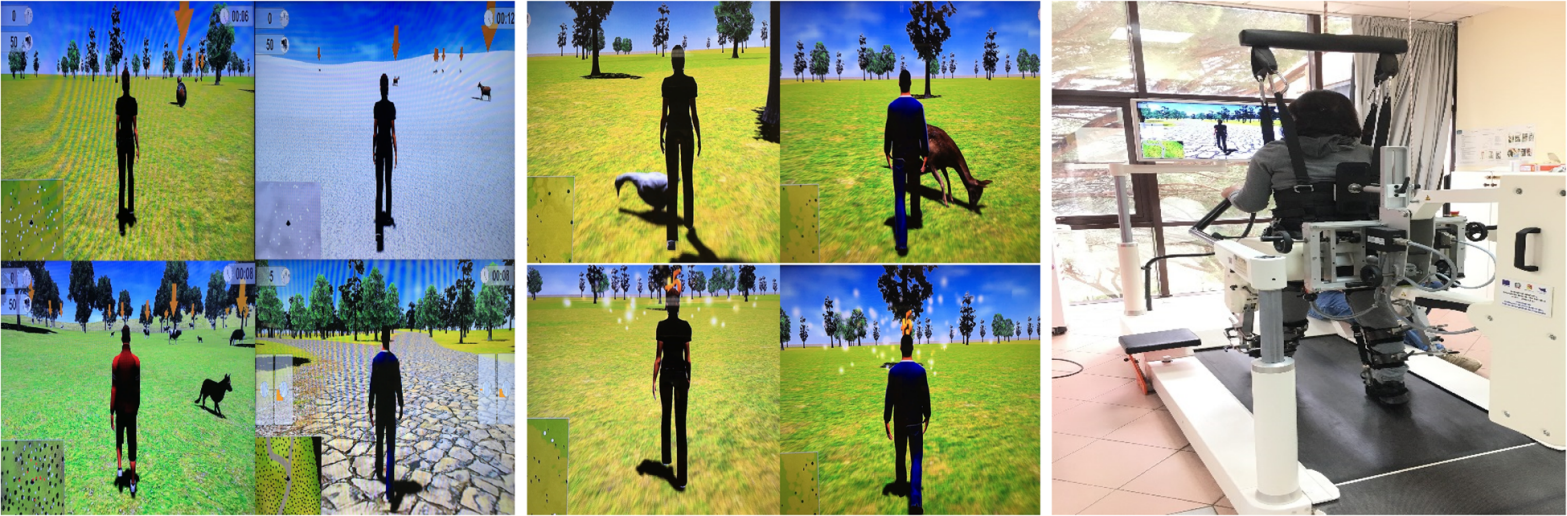
The virtual mirror (left), a sequence of the visual feedback (central), and combined visual-haptic modality (right)

Prior to the Lokomat training, all participants became familiarized with the Lokomat-Pro (RAGT + VR) or Lokomat-Nanos (RAGT-VR) (Hocoma AG; Volketswil, Switzerland), and had individually adapted their body weight support (BWS), leg guidance force (GF), and foot-lifting straps (that assisted ankle dorsiflexion for adequate toe clearance during the swing phase). Gait speed was set at a maximum of 1.8 m/s. Patients were asked to walk with their maximal effort. Then, patients were randomly assigned to RAGT + VR or RAGT-VR group. Both groups performed 40 Lokomat sessions (each lasting 40-45 min), five times a week, between 9^am^ and 11^am^. The RAGT + VR group received a visual feedback showing a VR run game where the patient had to collect or avoid objects, to motivate him/her to walk actively (Fig. 1). Each avatar’s leg movement corresponded to that performed by the patient. To this end, we employed an impedance-based control strategy [45, 46], which allows variable deviations from a given leg trajectory, thus making the orthosis feel compliant (instead of making the orthosis stiff, i.e., allowing only negligible deviations from the set trajectory). The Lokomat is equipped with potentiometers and force transducers, capable of providing feedback about joint movement and joint moment production, respectively. In the impedance-based control strategy, the joint moment acting on the subject’s leg (which is generated by the force transducers of the orthoses) is proportional to the angular deviation of the performed movement from the preprogrammed movement. Therefore, the patient perceives the stiffness of the orthosis based on the setting of the mechanical impedance value (i.e., zero impedance makes no feedback experienceable, whereas maximal impedance results in maximal stiffness, equal to the position controller). The stiffness of the orthoses was set to the lowest point where subjects reported feeling the inertia of the Lokomat segments (about 30% of the maximal Lokomat impedance).

On the other hand, the RAGT-VR group was not provided an avatar but a smile, indicating the goodness of the leg movements. The biofeedback of the Lokomat gait orthosis is based on the interaction torques between the participant and the orthosis. To this end, hip and knee linear drives are equipped with force sensors that measure the human-machine interaction forces at the hip and knee joints for stance and swing phase, which are required to keep the participant on a predefined gait trajectory. The weighting functions of the gait cycle were defined for each part so that the resulting biofeedback values increased for therapeutically desirable movements, i.e. hip and knee flexion-extension force. Thus, biofeedback force values (representing the physical activity of the participants) were positive when the patient was actively participating and negative when passively participating in Lokomat training (or when muscles were inappropriately or involuntary activated) [36]. We obtained an average biofeedback value for each joint in the hemiparetic leg.

## Virtual reality

The 2D VR set-up consisted of a 42-in. flat-screen placed in front of the Lokomat and a 7.1 Dolby Surround system (Fig. 1). The Lokomat device served as a multimodal feedback system: the human-machine interaction forces measured from the Lokomat device were used as an input device for the patient’s movements into the VR (i.e., to animate the motion of the human figure in VR at a 60 Hz refresh rate in real time on the screen – virtual mirror. There was no lag between motions of the subject and virtual figure) or the smiling face. The orthosis guided subject’s leg movements in the sagittal plane within individually adapted hip and knee joint trajectories. Lokomat potentiometers provided real-time information of the subject’s hip and knee angles. The measured joint angles were used to animate the subject’s human figure in the virtual mirror. The distance between the subject and projection screen was 1.5 m. Furthermore, since the run lane was not always straight, the VR running game used an asymmetrical physical activity of the legs to induce turning in the virtual environment. In particular, turning right and left was induced by increasing the activity of the contralateral leg of the desired direction and, respectively, decreasing the activity of the ipsilateral one. Lokomat device provided visual and acoustic feedback that reflected the interactions with objects represented in the virtual environment (e.g., the boundaries of the run lane, or objects to be avoided or collected). Further, Lokomat provided haptic feedback by the gait orthosis, so that the subjects using the device were provided with a haptic experience from proprioceptive (joint angles) feedback about their movements [47, 48].

The use of 2D displays, which are not as realistic as the true stereo 3D ones (full-3D VR), are akin to looking at a scene through a window and offer a limited sense of presence, potentially limiting the significance of our findings. We potentially provided a higher sense of presence by using depth cues, such as perspective, relative motion, occlusion, and aerial perspective, despite the use of a 2D VR [7].

### EEG recording and preprocessing

EEG was recorded using a Brain-Quick System (Micromed; Mogliano Veneto, Italy), from standard 19 electrodes headset according to the International 10-20 system (Fp1, Fp2, F7, F3, Fz, F4, F8, T3, C3, Cz, C4, T4, T5, P3, Pz, P4, T6, O1, O2, ground on the forehead), for 10 min while performing Lokomat training. To monitor eye movements, an electro-oculogram (EOG) with a bipolar montage (one pair of electrodes traced horizontal eye movements, a second pair the vertical ones) was also collected. EEG end EOG were sampled at 500 Hz, high pass filtered at 1 Hz using a zerophase FIR filter (order 7500) to minimize drifts, low pass filtered at 200 Hz (zerophase FIR filter order 36), and referenced to Cz [49]. An adaptive filter was applied to allow real-time filtering of signals recorded from EOG [50, 51].

Electrode impedance was kept below 5kΩ. During the entire EEG recording (as well as during the entire gait training), an experimenter checked for possible signs of drowsiness (e.g., abrupt worsening in gait performance, closed eyes, increase of proportion of theta and alpha activity in the eyes-open condition) [52], which were counted (given that monotonous gait pattern provided by RAGT may tend to induce sleepiness, thus decreasing arousal that negatively affects gait training progress). Patients were prohibited from drinking coffee, smoking, and change their bedtime during the three days prior EEG recording.

Infomax independent component analysis (ICA) was computed on the preprocessed EEG signal to decompose neural and artefactual sources [53–57]. In detail, ICA was computed two times. First, 500 ms-segmented EEG signals were removed if its probability distribution exceeded the average distribution by 5 ± SD. Then, ICA was computed to reject epochs based on the probability distribution of the IC projections. Thus, EEG segments were re-filtered (8-40 Hz) and a second ICA was computed a second time. The so-obtained IC were grouped into clusters using a k-means algorithm (based on the feature vector of dipole location, power spectra, and scalp map). The IC closest to the cluster centroid was remained for each subject, so to have equal contribution of each subject to the cluster-wise analysis.

### EEG analysis

EEG analysis consisted of the computation of the power spectral density (PSD) (using Welch’s Method) and the time-frequency analysis to evaluate Event-related-spectral-perturbations (ERSPs) for each IC [58].

EEG was segmented into 1.4 s epochs (−700;700)ms with regard to the heel strike (HS) (i.e., the first moment the foot comes into contact with the floor) [59], thus obtaining 428 epochs. About that, the force-sensing resistor of the Lokomat device detected the movement onset of both lower limbs, which was synchronized with the EEG data. Epochs were rejected by using an automatic artifact rejection method (epochs with values of [−100;100]μV, ≥5SD of the mean kurtosis value, ≥5SD of the mean probability distribution, drifts of ≥50 μV/epoch and with a R^2^ limit ≤0.3, spectra deviating from the mean by ±50 dB in the 0-2 Hz frequency window and by [−100;50]dB in the 20-100 Hz frequency window), visually inspection for artifacts, and if the power perturbation in the 20–40 Hz band deviated by +25 or -100 dB from the baseline at least for one IC [57, 60]. Rejection rate was 5%. This low rate is not surprising, given that it has been shown that scalp EEG recording during low-speed treadmill walking is not invalidated by excessive artefacts [61]. After this, the segmented data were time-warped and averaged together for all strides, so that initial affected-side heel strike, unaffected-side toe off, unaffected-side heel strike, affected-side toe off, and the subsequent affected-side heel strike occurred at the same times [49]. Spectrum analysis was carried using a standard fast Fourier transform (FFT) algorithm (Hanning-window, frequency resolution 0.7 Hz) within ϑ (4-7 Hz), μ (8–12 Hz), β (12–30 Hz), low-γ (Lγ) (31-45 Hz), and high-γ (Hγ) (46-70 Hz) bands [62, 63], and related to the phases of the gait cycle [55]. We opted to analyze these rhythms as it has been reported a different, specific role of each oscillation in sensory-motor pattern [27–35, 64]. For instance, there is evidence for a difference between the low and high α oscillations, which express action execution and observation, respectively [65, 66].

Single trial spectograms were computed and time-warped (thus aligning the time-points for right and left heel strike) over trials using a linear interpolation function to generate gait cycle ERSPs (i.e., epochs were based on the heel strike events, being the unaffected-side, the affected-side, and the next unaffected-side heel strike time-warped to 0, 50%, and 100% of the gait cycle, respectively). Relative changes in spectral power were obtained by averaging the difference between each single-trial log spectogram and baseline (the mean IC log spectrum over all gait cycles per training) [49]. To visualize significant ERSP changes, deviations from the average gait cycle log spectrum were computed with a bootstrap method [56, 57]. For statistical concern, bandwidth ERSP of each IC were averaged within each 10% of the gait cycle (10-point ERPS curve, frequency resolution 0.7 Hz) [67]. The average log spectrum for all movement cycles was subtracted from the log spectrogram for each movement cycle. We thus calculated the resulting PSD changes from this baseline (defined as the percentage decrement, event-related desynchronization –ERD- and increment, event-related synchronization –ERS- as a function of the percentage of the normalized gait cycle) [53] for each band and electrode-group of interest (with regard to the areas of activation of MNS reported in the literature [68], i.e., ipsi and contralesional frontal -Fp1/F7/F3, Fp2/F8/F4-, central -T3/C3, T4/C4- and parieto-occipital -T5/P3/O1, T6/P4/O2) [69–73].

### Source localization

The source localization approach allows examining brain activities in various sources at different temporal phases of motor control. Because of high temporal resolution of the EEG signals, brain activities before and after the movement onset can be localized in order to distinguish cortical activities related to both motor planning (movement preparation) and motor execution (corticospinal pathway activation). The Estimation of Current Densities was carried by using Low Resolution Brain Electromagnetic Tomography (LORETA; free release of LORETA-KEY alpha-software) [63, 74–76]. The main components detected with the ICA (signal-to-noise ratio   >  1) were chosen for the source reconstruction. The distributed current density model (LORETA) with L1 norm method (based on the Montréal Neurological Institute (MNI) brain MRI) was then applied to the ICA data [77, 78]. The sources were constrained to the reconstructed layer of the folded cortex [79, 80].

### Outcome measures

The primary endpoint, with respect to VR efficacy in post-stroke condition, was the proportion of patients achieving a 20% improvement in lower limb gait and balance at the end of the training, as measured by the Rivermead Mobility Index (RMI), the Tinetti Performance Oriented Mobility Assessment (POMA), and the gait cycle-related ERSPs. Indeed, a 20% improvement correspond to a significant minimal detectable change in RMI [81] and POMA [82]. According to previous work on assisted gait training in post-stroke patients, these changes are paralleled by EEG signal modification of at least 20% to be significant [83, 84].

As secondary outcomes, we considered the global MAS score derived from the muscles of hip, knee, and ankle, the Hamilton Rating Scale for Depression (HRS), the hip and knee flexion/extension force measured by the RAGT device, the extent to which a patient felt him/herself entrained in the VR training (reported on a visual analogue scale -VAS- ranging from zero -not at all- to ten -very much), and the mean of the episodes of drowsiness.

### Statistical analysis

The normal distribution of the data was evaluated with the Kolmogorov-Smirnov test. Baseline data were compared between the two groups using a Student *t-*test for continuous variables if data were normally distributed, whereas a Mann-Whitney U test was used for non-normally distributed ordinal scale. Likewise, Wilcoxon test, Mann-Whitney U test, or t-test were used for within-group and between-group comparisons, depending on the types of data measurements.

The ERD/ERS changes for each frequency band were assessed by means of three-way ANOVA for repeated measures, employing the factor *time* (two levels: T_PRE_ and T_POST_) and *electrode-set* (three levels: frontal, central, and parieto-occipital) as within-subject factors, and *group* (two levels: RAGT + VR and RAGT-VR) as between-subject factor. Based on the significance of the F-value, *post-hoc* paired-sample *t*-tests were carried out to assess the significance of interactions (Bonferroni correction). A *p*-value <0.05 was considered significant.

BWS and GF were included as covariates in the ANOVA analysis. In fact, it has been reported that RAGT training usually implies a steady progression of BWS and GF across the training program, so it is necessary to update and analyze these parameters in relation to the assessment of the outcomes [85]. In fact, both BWS and GF can influence spatiotemporal movement characteristics, thus affecting functional gait pattern. Indeed, finely tuning BWS and GF may somehow improve possible spatiotemporal gait asymmetries. On the other hand, missing the correction of these parameters augments inter-limb gait asymmetry for an extended duration in people with stroke [86]. Besides the factor *electrode-set* (which was employed in the ANOVA analysis to carry the spatio-temporal analysis of EEG signals at scalp level), we also added the factors *lesion localization* as covariate in the ANOVA analysis (according to the localization within left or right frontal, parietal, occipital, and temporal lobe). Such factor was added to augment inter-subject evaluation, as the sample was non-homogeneous for stroke localization, which can affect EEG signals beyond the overhead electrodes [87], also influencing both motor deficit degree and recovery [88].

ERSPs were computed in each frequency range for RAGT + VR and RAGT-VR using the average gait cycle log spectrum computed from the RAGT-VR as common baseline. The gait cycle was divided into in two stationary (S1, 10–30%, and S2, 60–80%) and two transition phases (T1, 30–60%, and T2, 80–10%). The stationary phases correspond to the midstance (10–30%), initial swing (60–70%), and midswing phases (70–90%), whereas the transition phases correspond to the terminal stance (30–50%), preswing (50–60%), terminal swing (90–100%), and loading response (0–10%) [89]. An ANOVA for repeated measures with the factors *time* (in relation with the gait cycle phases) (eight levels: two PRE and POST stationary and two PRE and POST transition phases), *electrode-set* (three levels: frontal, central, and parieto-occipital), and *group* (two levels: RAGT + VR and RAGT-VR) was computed for each frequency band. Multiple comparisons were corrected controlling for false discovery rate (*p* < 0.05) [90]. Sphericity assumption violations were Greenhouse-Geisser corrected.

## Results

### Participant flow

**Fig. 2:**
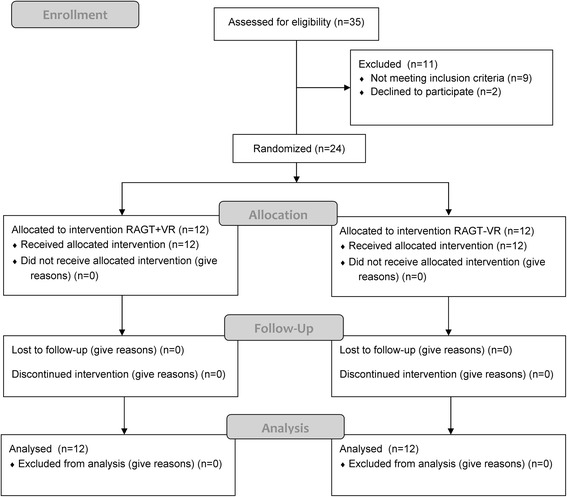
The CONSORT flow diagram

We summarized in the CONSORT flow diagram (Fig. 2): the numbers of participants who were randomly assigned, who received the intended treatment, and who were analyzed for the primary outcome; the losses and exclusions during periods of recruitment, randomization, and follow-up. All treated patients completed the robotic training without reporting any side effect (Fig. 2). The main analysis focused on the consequences of the avatar observation on the RMI, POMA, and gait-related ERS/ERD, and involved all the patients who were randomly assigned to the two groups. Twelve patients were enrolled in each group (RAGT + VR: mean age 60 ± 4 years, 7 males and 5 females, disease duration 8 ± 2 months; RAGT-VR: mean age, 63 ± 6 years, 7 males and 5 females, disease duration 8 ± 2 months) (Table 1). There were no significant differences concerning any parameter between the two groups. Mean and standard deviation values of all outcome measures are reported in Table 1.

### Clinical and kinematic data

RMI improved more in the RAGT + VR than the RAGT-VR group, whereas POMA improved only in the RAGT + VR group (Table 2). HRS score equally decreased in both groups. MAS did not change significantly in both groups. VAS improved only in RAGT + VR group (Table 2). Knee force showed a greater improvement in RAGT + VR than RAGT-VR group, whereas hip force improved only in RAGT + VR group (Table 2).

**Table 2:** Mean values (±SD) of clinical outcomes measure after RAGT ± VR. Within- and between-group comparisons, with confidence interval (95%) (CI), are reported. Not reported data are non-significant

parameter	Group	PRE	POST	Within-group	Between-group	CI (95%)
RMI	RAGT + VR	8 ± 1	14 ± 1	<0.001	0.001	1.2 to 7.6
	RAGT-VR	7 ± 1	9 ± 1	0.01		
POMA	RAGT + VR	17 ± 3	23 ± 3	0.001	0.01	1.2 to 7.6
	RAGT-VR	12 ± 4	15 ± 4			
MAS	RAGT + VR	2 ± 0.5	2 ± 0.5			
	RAGT-VR	2 ± 0.5	2 ± 0.5			
HRS	RAGT + VR	11 ± 3	7 ± 3	0.01		
	RAGT-VR	13 ± 3	10 ± 3	0.02		
VAS	RAGT + VR	6 ± 1	8 ± 1	<0.001	0.01	1.3 to 15.3
	RAGT-VR	5 ± 1	6 ± 1			
drowsiness episodes	RAGT + VR	5 ± 1	2 ± 1	<0.001	0.009	1 to 6.9
	RAGT-VR	5 ± 1	5 ± 1			
Hip force	RAGT + VR	36 ± 7	42 ± 3	0.01	0.02	−6.2 to −3.8
	RAGT-VR	34 ± 6	38 ± 10			
Knee force	RAGT + VR	31 ± 8	47 ± 6	<0.001	0.02	
	RAGT-VR	30 ± 7	36 ± 3	0.04		

*Legend*: RMI Rivermead Mobility Index; POMA Tinetti Performance Oriented Mobility Assessment; MAS Modified Ashwort Scale; HRS Hamilton Rating Scale for depression; VAS visual analogic scale

### Electrophysiological data

**Fig. 3:**
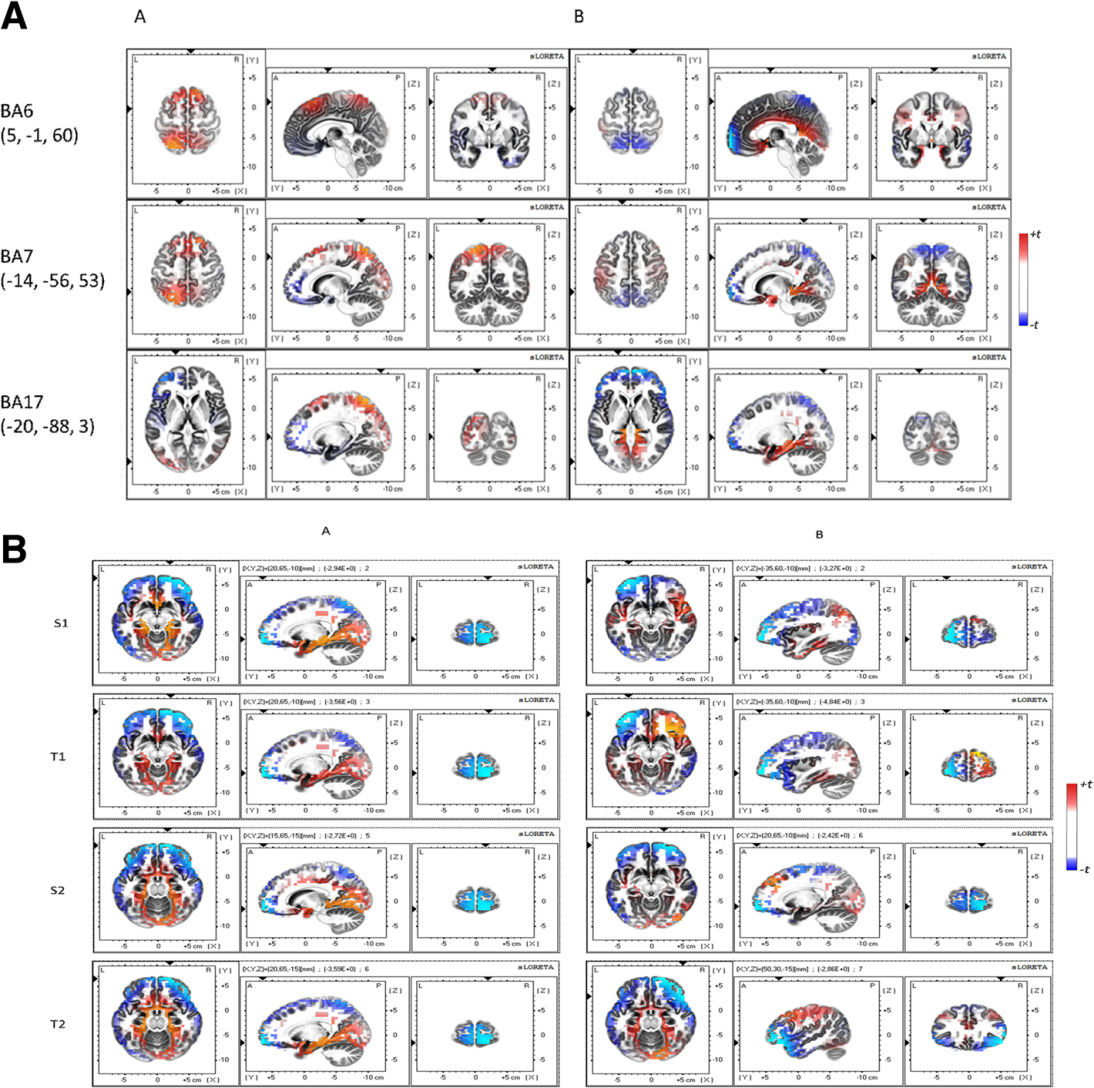
T_PRE_-T_POST_ changes in area activation during the entire gait cycle (panel **a**) or each phase of gait cycle (S1, T1, S2, and T2) (panel **b**) as identified by LORETA in RAGT + VR (**a**) and RAGT-VR (**b**) groups. Lesioned area is plotted on the left hemisphere. The colors refer to the voxel-by-voxel t-tests values for T_PRE_-T_POST_ changes (see Table 3)

We found three main areas of brain activation that were more evident in the RAGT + VR group as compared to the RAGT-VR group across the gait cycle (Fig. 3a): (1) BA6 (Tailarach coordinates -x,y,z- 5, −1, 60); (2) BA7 (−14, −56, 53); and (3) BA17 (−20, −88, 3).

**Fig. 4:**
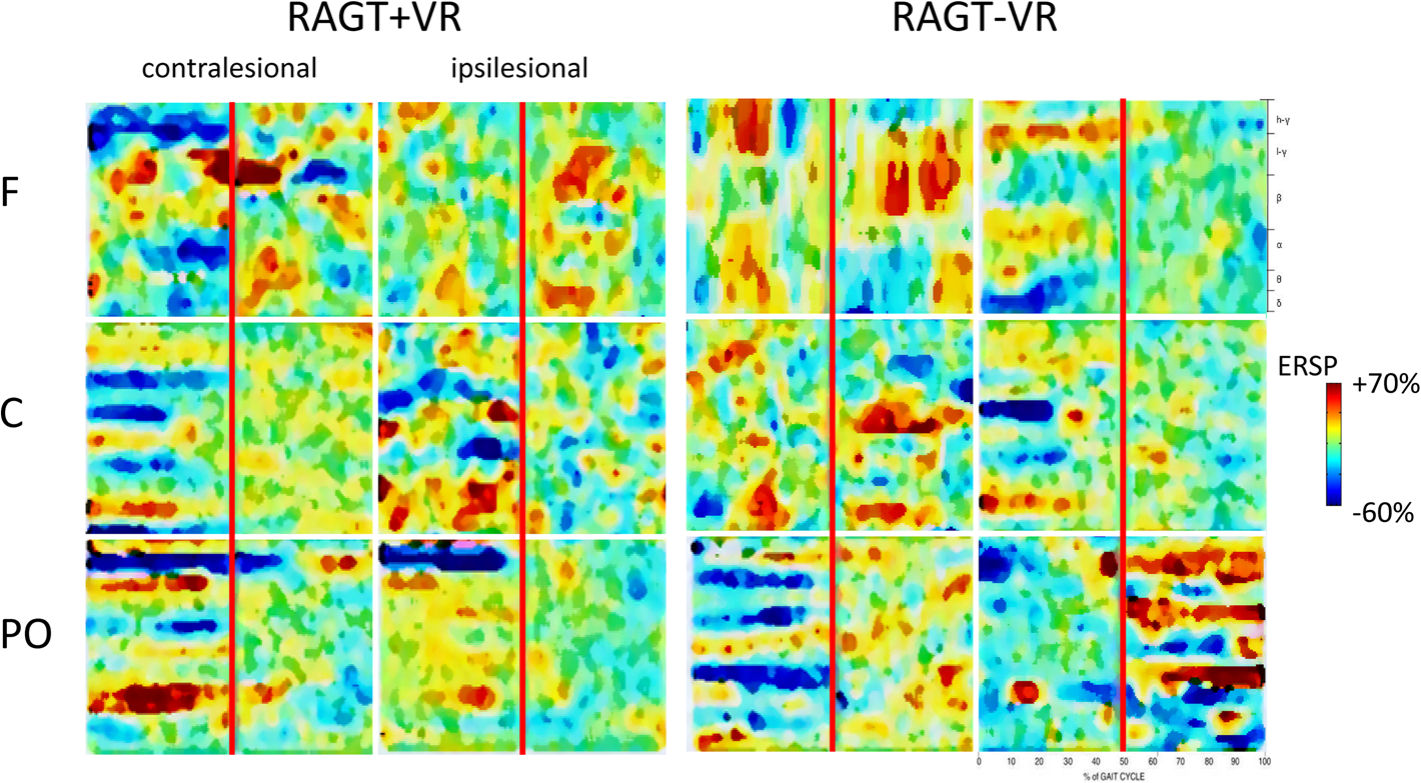
Average T_PRE_-T_POST_ changes in gait event-related spectral perturbation (ERSPs) in RAGT + VR (**a**) and RAGT-VR (**b**) groups. Non-significant differences relative to the full gait cycle baseline (*p* ≤ 0.05) are masked in green (see Table 4)

**Fig. 5:**
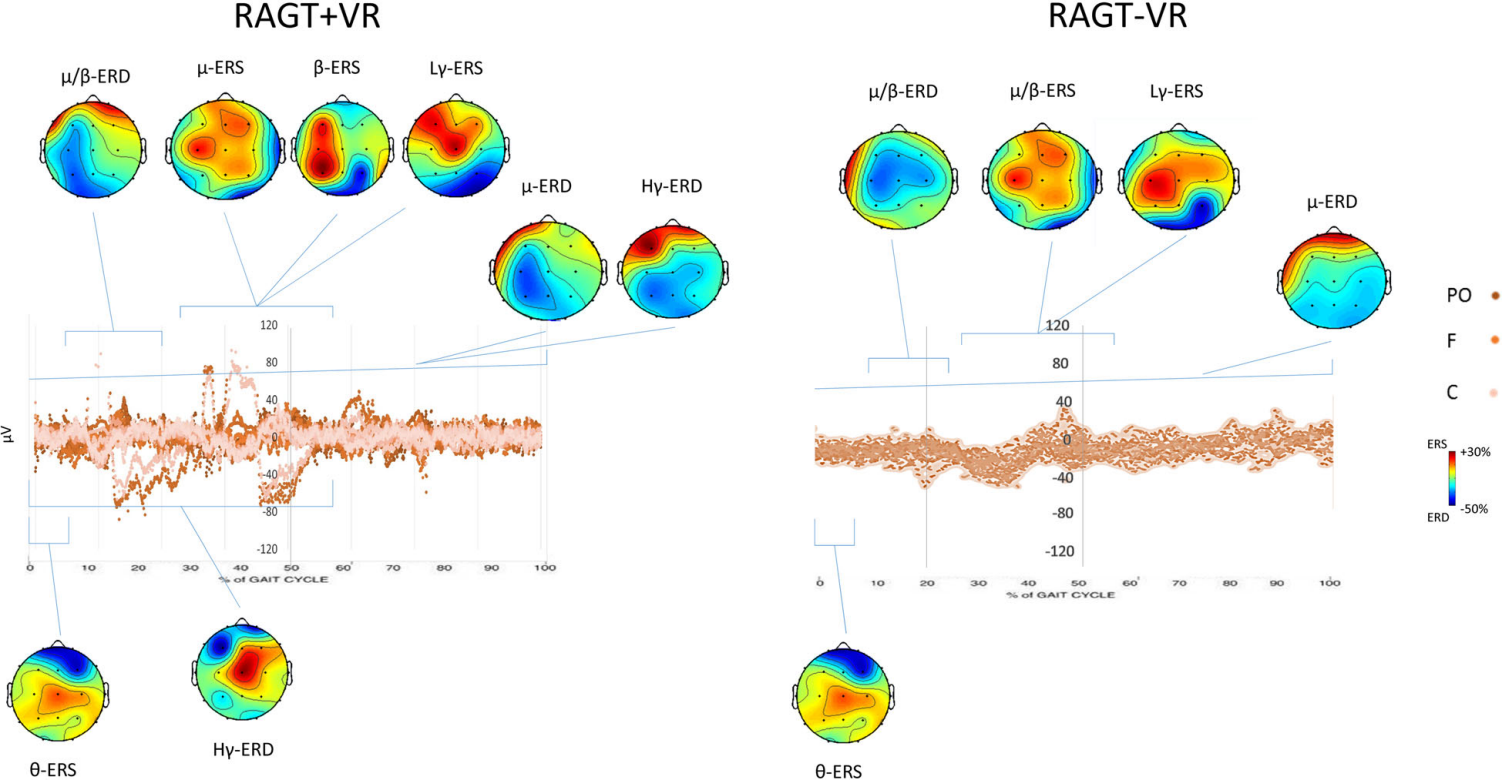
Average changes at T_POST_ as compared to T_PRE_ in scalp ERP projections relatively to the full gait cycle. The left and right hemispheres plots correspond to the affected and unaffected ones, respectively. ERS and ERD are masked in red and blue tones, respectively, whereas non-significant differences are in green (see Table 5)

Concerning frontal activation (Fig. 3b; Table 3), RAGT + VR induced significant μ/β ERSP changes from baseline to S1, T1, and S2 phase of the gait cycle, as reflected by the significant interaction between gait phases and training for gait cycle related modulations concerning RAGT + VR group (Table 4). In particular, we observed a frontal ERD before the heel strike, followed by a frontal ERS during the heel strike (Fig. 4; Table 4). This changes also presented a significant difference in scalp projections between the trainings (Table 5; Fig. 5), with a greater magnitude in RAGT + VR group.

**Table 3:** Significant different LORETA activation (ANOVA F and *p* values) during gait cycle at T_POST_ as compared to T_PRE_ (*post-hoc p-*value). Not reported data are non-significant

*t × g × e*	RAGT + VR						RAGT-VR					
	*t × e*		T_PRE_-T_POST_ differences related to gait cycle phases				*t × e*		T_PRE_-T_POST_ differences related to gait cycle phases			
			S1	T1	S2	T2			S1	T1	S2	T2
21, <0.001	28, <0.001	C	<0.001	<0.001	0.03	<0.001	8, 0.01	C	<0.001	<0.001	<0.001	<0.001
		F	<0.001	<0.001	<0.001	<0.001		F	<0.001	<0.001	<0.001	<0.001
		PO	0.004	0.005	<0.001	<0.001		PO	<0.001			

*Legend*: t time, g group, e electrode, C central, F frontal, PO parieto-occipital; S1 first stationary phase; S2 second stationary phase; T1 first transition phases; T2 second transition phase

**Table 4:** Summarizes the ANOVA findings concerning PRE-POST group differences of ERSP (F, p) (top) and the *post-hoc p-*values (t, p) (bottom) related to each phase of gait cycle. Not reported data are non-significant

band	*t × g × e*	RAGT + VR				RAGT-VR		
		*t × e*	C	F		*t × e*	C	F
μ	8.6, <0.001	77, <0.001	71, <0.001	50, <0.001		16, <0.001	3, 0.007	5, <0.001
β	15, <0.001	72, <0.001	87, <0.001	55, <0.001		39, <0.001	8, <0.001	9, <0.001
Lγ	9.2, <0.001	66, <0.001		53, <0.001		12, <0.001		25, <0.001
Hγ	28, <0.001	37, <0.001		12, <0.001				
ϑ	8.9, <0.001	57, <0.001	51, <0.001			10, <0.001	18, <0.001	
band	electrode	group	PRE-POST differences related to gait cycle phases					
			S1	T1	S2	T2		
μ	C	RAGT + VR	3.3, 0.01	−14, <0.001	3.4, 0.01			
		RAGT-VR	2.4, 0.04	−6.3, 0.001	3.3, 0.01			
	F	RAGT + VR		5, 0.002	4.5, 0.004			
		RAGT-VR		3.6, 0.01	2.7, 0.03			
	PO	RAGT + VR	-3.4, 0.01	−5, 0.002	−3.4, 0.01	−3.6, 0.01		
		RAGT-VR	-3.3, 0.01	4.5, 0.002	−3.3, 0.01	−2.7, 0.03		
β	C	RAGT + VR	-26, <0.001	6, 0.001	4.8, 0.003			
		RAGT-VR	-2.9, 0.02	3.9, 0.007	2.9, 0.02			
	F	RAGT + VR	-24, <0.001	8.3, <0.001	5.3, 0.002			
		RAGT-VR	-3.5, 0.01	3.5, 0.02	3.5, 0.01			
ϑ	C	RAGT + VR	5.1, 0.002					
		RAGT-VR	3, 0.02					
Hγ	F	RAGT + VR	-3.5, 0.01	−2.6, 0.03	−5.2, 0.002			
		RAGT-VR						
	PO	RAGT + VR	-2.8, 0.02	−4.4, 0.004	−7, <0.001	−4.5, 0.003		
		RAGT-VR						
Lγ	C	RAGT + VR		6, 0.001	5.1, 0.002			
		RAGT-VR		3.5, 0.02	3.8, 0.008			
	F	RAGT + VR		15, <0.001	13, <0.001			
		RAGT-VR		4.9, 0.003	4.9, 0.003			

*Legend*: t time, g group, e electrode, C central, F frontal, PO parieto-occipital; S1 first stationary phase; S2 second stationary phase; T1 first transition phases; T2 second transition phase

**Table 5:** Summarizes the significant PRE-POST differences (t, p) of scalp projections. Not reported data are non-significant

band	RAGT + VR			RAGT-VR					
	C	F	PO	C		F		PO	
μ -ERD	5 < 0.001		11, <0.001	6, <0.001				4, <0.001	
β -ERD	10, <0.001		4, <0.001	6, <0.001		6, <0.001		3, <0.001	
μ -ERS	5, <0.001	8, <0.001		13, <0.001		10, <0.001			
β -ERS	4, <0.001	13, <0.001							
ϑ -ERS	5, <0.001		4, <0.001	6, <0.001				4, <0.001	
Lγ -ERS	7, <0.001	4, <0.001		6, <0.001		22, <0.001			
Hγ -ERD		4, <0.001	4, <0.001						

*Legend*: t time, g group, e electrode, C central, F frontal, PO parieto-occipital

μ/β changes were paralleled by a significant Lγ-ERS around the heel strike in both the groups and an Hγ-ERD from S1 to S2 (i.e., ending soon after the heel strike) only in RAGT + VR. Detailed statistical data of the main interactions and effects, and the temporal ERSP changes across the gait cycle are summarized in Tables 4 and 5 and Figs. 4 and 5.

Concerning central areas activation (Fig. 3b; Table 3), we found a significant μ/β ERD/ERS at S1, T1, and S2, a brief ϑ-ERS in the S1 phase, as revealed by the ANOVA and the post-hoc tests (Tables 4 and 5; Figs. 4 and 5) showing that spectral power in μ/β band was significantly reduced in T1 and increased in S1, and this was more evident in RAGT + VR than RAGT-VR.

Finally, parieto-occipital activation (Fig. 3b; Table 3) was characterized by a significant difference for μ and Hγ (ERD) between the groups during the entire gait cycle (Tables 4 and 5; Figs. 4 and 5). Post-hoc tests showed that scalp projections were significantly strengthened in RAGT + VR as compared to RAGT-VR.

### Clinical-electrophysiological correlations

We observed a significant correlation between RMI and POMA score improvement and central β-ERS (*r* = 0.895, *p* = 0.001 and *r* = 0.570, *p* = 0.04, respectively) and frontal Hγ-ERD magnitude (*r* = −0.831, *p* = 0.003 and *r* = −0.615, *p* = 0.04, respectively). Moreover, the improvement in hip force was significantly correlated with frontal Hγ-ERD magnitude (*r* = −0.802, *p* = 0.004). Clinical-demographic characteristics (age, gender, disease duration, comorbidity) did not influence the primary outcomes.

## Discussion

The main finding of our pilot study consists in the more evident activation of premotor, precuneus, and associative visual areas in the RAGT + VR group as compared to RAGT-VR group.

All the patients belonging to RAGT + VR showed a significant decrease of central μ/β power during the phase preceding the heel strike, followed by a power increase (as shown by the gait cycle phase dependent ERSP modulation), thus indicating higher neuronal activation [91]. Importantly, we observed that the stronger the μ/β ERSPs were, the higher the clinical amelioration. Given that these ERSPs are a marker of activation and deactivation/inhibition of sensorimotor areas concerning motor planning, postural stabilization, and the prediction of potential actions [92–102], our findings suggest the importance of enhancing μ/β ERSPs to foster locomotor training. In addition, to monitor these brain activations would allow a better patient-tailored walking training.

The novelty of our study is the significant fronto-parietooccipital Hγ-ERD and parietooccipital α-ERD only in the RAGT + VR group. The premotor-parietooccipital desynchronization of γ-oscillations is thought to be a marker of activation of sensorimotor and visuo-spatial associative areas concerning motor planning and selective muscle activation [91, 100, 102–112] even during active and passive RAGT [67].

We also found that the magnitude of γ-band modulation was significantly correlated with the clinical amelioration and the improvement in muscle strength, and it was paralleled by a more selective μ/β-band modulation concerning either the temporal patterns of activation across the gait cycle or the hemispheric distribution of ERSPs. We may argue that VR may induce a functional fronto-parietooccipital α/γ-band activation that, in turn, allows a more efficient motor planning and execution, as shown by a stronger and selective modulation of μ rhythm across the phases of gait cycle. Such a selective modulation allows the patient to complete better the gait training (e.g., to better steer, avoid objects, and keep the line during walking) [113]. These data are in keeping with the role of the premotor areas in planning limb movements [114] and initiating and adapting gait [115–118], and of the parieto-occipital cortex in spatial attention, decision making, sensorimotor integration, and movement planning in visually guided movements under both feedforward and feedback control [119–124]. The specific entrainment of γ rhythms when observing a human avatar may depend on a different entrainment of visuomotor networks as compared to the control condition (RAGT-VR). According to the canonical microcircuit model [125], the superficial pyramidal neurons generating γ-responses act as a dynamic filter on the visual inputs, thus affecting both the configuration of γ-oscillations (depending on the stimulus properties, including movement, contrast, localization, and size of visual cues) and the μ and β band output dynamics (which are generated by deeper pyramidal neurons) [126–128]. The use of an avatar may have thus specifically increased the frontal-posterior γ oscillations. The parieto-occipital α-ERD may be instead linked to basic visual processing. In addition, it has been reported that μ/γ ERSP provided by VR feedback is related to the participants’ monitoring of their own movements [129–131]. We can therefore hypothesize that these ERSPs in fronto-parietooccipital regions during the observation of performed movements and during visually-guided gait adaptation task potentially express the activation of the MNS.

One could argue that Hγ ERSPs may purely reflect motor activation and not specific cognitive processes related to VR, given that γ-band ERSPs express also a higher cortico-muscular connection during ambulation [132–134]. Nonetheless, this concern sounds unlikely, since BWS and DGF, which both change muscle activity [135, 136], were individually adapted in all patients. Consequently, Lγ band (which is instead strongly related to motor activity level) [132, 133, 137] was similar in the two groups, despite BWS and DGF individual adaptation, whereas Hγ ERSPs reflected the presence of VR rather than to motor practice.

A brief ϑ-ERS (at the beginning of the gait cycle) was present in both groups. It is hypothesizable that ϑ-ERS in a non-specific event during gait and it is probably related to the sensorimotor area demand, the basic locomotor control, and the timing of muscular activation patterns [138, 139].

ERSPs were lateralized in the affected hemisphere in the RAGT + VR group but not in the group RAGT-VR, despite the lesion localization was similar in both the groups, with the exception of parieto-occipital ERSPs, which were bilateral in both groups, as formerly reported [140]. In fact, visuomotor information processing is distributed symmetrically during walking [140], except for some specialized areas located in the right hemisphere, which are crucial for the closed-loop aspects of the movements depending on the sensory feedback [141]. We may argue that the bi-hemispheric distribution of ERSPs in the RAGT-VR may depend on a dysfunctional reshape of interhemispheric connectivity, which was instead recovered, at least partially, in the RAGT + VR group [142–147].

As limiting factors in our work, we have to acknowledge that patients were provided with objects appearing in different corners of the screen during RAGT + VR. This fact may force eye-movements planning, which is expressed by a decreased α/β power within parietooccipital regions [148]. Nonetheless, the extent of difference in brain activation between RAGT + VR and RAGT-VR is sufficiently high to exclude a biasing effect of the activity related to saccades on our data. Moreover, it has been shown that first-person perspective is superior to third-person perspective VR [149, 150], owing to an enhanced feeling of agency [151]. We could speculate that the increased performances might have induced a greater feeling of agency in the third person perspective. However, studies comparing first- and person perspective are needed to confirm this issue.

Further, the clinical improvement we reported may also depend on factors not directly related to the ERSPs, including a stronger motivation for active participation in the movement provided by the VR [152], as suggested by the few episodes of drowsiness and the high sense of entrainment in the VR setting. A stronger motivation is, however, of notable importance, given that it allows the patient to exercise more regularly, precisely, and intensely [153–156] and, at least indirectly by enhancing the voluntary drive, to improve motor planning, learning and execution [157, 158]. Thus, our results show anyway the possible benefit of goal directed walking tasks that recruit brain areas involved in motor planning, learning and execution by using VR.

Finally, we employed a relatively low-speed RAGT, which could have affected the timing of muscle activation and amplitude, thus potentially reducing the level of sensorimotor cortex activation. Nonetheless, we preferred to adopt a low-speed RAGT to avoid excessive EEG contamination due to movement artefacts.

In conclusion, our findings suggest that VR feedback during RAGT elicits stronger cortical activations within the fronto-parietooccipital areas potentially belonging to the MNS, and involved in motor intention and planning. These activations were paralleled by an evident improvement in walking ability. We may thus argue the use more demanding and interactive task during RAGT by using VR may be of benefit to the patients with stroke. Moreover, monitoring the EEG in this context allows clinicians to realize better patient-tailored rehabilitative approaches.

## Abbreviations

BWS: Body weight support
EEG: Electroencephalographic
EOG: Electro-oculogram
ERD: Event-related desynchronization
ERS: Event-related synchronization
ERSP: Event-related-spectral-perturbation
FFT: Fast Fourier transform
GF: Leg guidance force
HRS: Hamilton Rating Scale for Depression
HS: Heel strike
ICA: Independent component analysis
LORETA: Low Resolution Brain Electromagnetic Tomography
MAS: Modified Ashworth Scale
MNS: Mirror neuron system
MRC: Muscle Research Council
MRI: Magnetic resonance imaging
POMA: Tinetti Performance Oriented Mobility Assessment
PSD: Power spectral density
RAGT: Robot-assisted gait training
VAS: Visual analogue scale
VR: Virtual reality
